# A Piston-Rotaxane with Two Potential Stripes: Force Transitions and Yield Stresses

**DOI:** 10.3390/molecules181113398

**Published:** 2013-10-30

**Authors:** Edith M. Sevick, David R.M. Williams

**Affiliations:** 1 Research School of Chemistry, Australian National University, Canberra, ACT 0200, Australia; 2 Department of Applied Mathematics, Research School of Physical Sciences and Engineering, Australian National University, Canberra, ACT 0200, Australia; E-Mail: D.Williams@anu.edu.au.

**Keywords:** rotaxane, piston-rotaxane, one-dimensional gas, stripes, stations

## Abstract

We examine a rod piston-rotaxane system, where the positions of several mobile rings on the axle are controlled by an external force acting on one of the rings. This allows us to access the translational entropy of the rings. For a simple rotaxane molecule with an axle that has uniform ring-axle interactions along its length, the molecule behaves like a miniature piston filled with a one-dimensional ideal gas. We then examine the effect of two stripes on the axle, having different ring-axle interactions with the mobile rings, so that one section is of high energy (repulsive) for the rings and another section is of lower energy (or attractive). This kind of rotaxane can exhibit rapid changes in displacement or force, and in particular, this molecule can exhibit a yield stress in which the piston suddenly compresses under a small increase in the applied force.

## 1. Introduction

Rotaxanes have rightly received considerably attention from synthetic chemistry community [1,2,3]. However, the material properties and physics of these systems are relatively unexplored. There are thus many novel problems and scenarios, with fascinating physics, which are yet to be solved. Some progress has however been made. Early work focussed on so-called slip-link [4], or sliding polymer, layers [5,6] in which there is one ring per axle. More recent work has examined cases where there is more than one ring per axle, particularly for piston-rotaxanes for rod-like axles [7]. In these systems, one of the rings is connected to an external piston, which controls its position, hence allowing the external agent to access the translational entropy of the rings along the axle. There has been some interest in the effect of flows and forces on these [8] and their dynamics [9]. Some work has also been done with flexible polymers as the backbone [10] and on gels [11]. What has been emphasised in recent work is the entropic effect of mobile rings on molecular behaviour. If the rings are mobile along the backbone, then the system represents something close to a one-dimensional gas [7,12,13]. If one can change the “volume” of this gas, *i.e.*, the length of the axle accessible to the rings, by using a piston attached to one of the rings, one can generate novel materials that rely on the pressure of their mobile rings to resist imposed forces.

These systems also have a unique advantage to the physicist, in that their low-dimensionality means that they can be modelled simply and often “exactly”.

**Figure 1:**
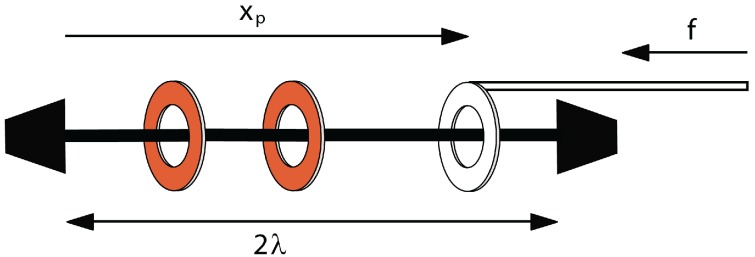
A simple piston-rotaxane. The axle has a length of 2λ. Here, two mobile rings on the left are compressed by the one piston ring on the right. The position of the piston ring is given by $x_{p}$, and it has a force, *f*, applied to it. The system behaves approximately as a one-dimensional ideal gas.

**Figure 2:**
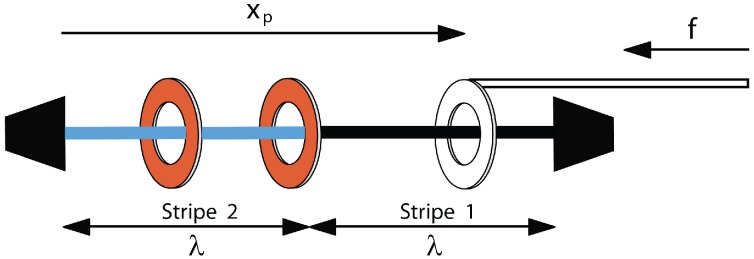
A piston-rotaxane with two potential stripes, each of a length of *λ*. The system behaves approximately as a one-dimensional gas, but the mobile rings now interact with the axle via a potential.

The simplest kind of system in this category is the piston-rotaxane (Figure 1), where *n* mobile rings are controlled by one ring attached to an external piston. The physics of this simple system was explored in [7]. Here, we consider a similar, but slightly different, system in which the axle is not uniform and the rings interact with the axle via a potential, *U*, which varies along the axle. This is motivated by two things. First, in experiments, one is often confronted by an axle, which has binding stations along it. Second, we can show that this system exhibits some interesting physics. In particular, here, we look at a striped axle (Figure 2), with only two equal-length stripes, one of high energy and the other of low energy. This system exhibits transitions as the piston is compressed and, in some cases, will show a yield stress in which the extension varies discontinuously as a function of the force.

A technical point needs to be emphasised at the start. We consider only the case of thermal equilibrium, so that ordinary equilibrium statistical mechanics can be used. This allows us to employ the machinery of partition functions and free energies to make predictions. If experiments are done on short time-scales, then non-equilibrium effects will appear. These are certainly interesting, but are beyond the scope of this paper (for an example of non-equilibrium effects in this piston-rotaxane with a uniform axle, see [9]).

It should also be noted that there are two distinct kinds of experiments that can be performed when compressing a system. In one, the compression is a given independent variable, controlled from the outside, and what is measured is the average force. In the other, the force is the independent variable, and the average extension is measured. For some common macroscopic systems, like an ideal gas in a container, these two experiments would give identical results. We know that $PV=nk_{B}T$, irrespective of whether we control *P* or *V*. For other systems, and in particular, those that show instabilities or contain very few particles, the two kinds of experiments show very different results.

The paper is organised as follows. We first review some simple results for a piston-rotaxane without any backbone potential; this is a purely entropic piston-rotaxane. We then look at the case of a striped system (with two stripes), in which the piston displacement is the independent variable, first with only one mobile ring and, then, with *n* mobile rings. Then, we examine the case in which we have force as the independent variable. Both these systems exhibit sharp transitions, but their nature is somewhat different. The detailed calculations can become somewhat involved and are relegated to the Appendix.

## 2. The Simple Piston-Rotaxane

Consider a rigid straight axle of a length of 2λ with *n* mobile rings, all to one side of another ring, the piston ring, which is attached to another rod (Figure 1). This is the piston-rotaxane [7]. We will assume for simplicity that the rings slide freely along the axle and do not interact with it or with each other, except that they cannot pass through the axle or each other. We also assume that the rings take up no volume, *i.e.*, the excluded volume is zero. This assumption can be easily relaxed, as was done previously [7,12], but relaxing it changes the physics very little.

We let $x_{p}$ be the distance of the piston ring from the far end of the axle. This system can be analysed exactly by calculating the partition function, which is the sum over all possible states of the Boltzmann factor, $e^{-\beta U}$, where $\beta \equiv 1/k_{B}T$ and *U* is the energy of the system. As we assume no interactions between any of the rings and the axle, *U* can be set to zero.

For a system of only one mobile ring, the partition function can be written as:

(1) $$Z_{1x}=\int_{0}^{x_{p}}dx_{1}e^{0}=x_{p}$$

If we have *n* identical rings, the partition function is just [12]:

(2) $$Z_{nx}=\frac{1}{n!}Z_{1x}^{n}$$

The free energy is then $F=-k_{B}T\ln\left(Z_{nx}\right)$, and the average force felt by the piston in equilibrium is

(3) $$\langle f\rangle =-\frac{\partial F}{\partial x_{p}}=\frac{k_{B}T}{Z_{nx}}\frac{\partial Z_{nx}}{\partial x_{p}}=nk_{B}Tx_{p}^{-1}$$

which is just the one-dimensional equivalent of the ordinary ideal gas law, PV=NRT.

If we allow the force, *f*, imposed on the piston to be the independent variable, we have a slightly more complicated calculation. Such a force is equivalent to a potential $U_{p}=fx_{p}$ acting on the piston ring, which can now take up any position along the rod of a length of 2λ. The partition function is then:

(4) $$Z_{nf}=\int_{0}^{2\lambda }dx_{p}e^{-\beta fx_{p}}Z_{nx}\left(x_{p}\right)={\left(\beta f\right)}^{-(n+1)}\left[1-e^{-2\beta f\lambda }e_{n}\left(2\beta f\lambda \right)\right]$$

with $e_{n}\left(x\right)\equiv \sum_{k=0}^{n}\frac{x^{k}}{k!}$.

The average position of the piston ring is then:

(5) $$\langle x_{p}\rangle =-\beta^{-1}\frac{\partial \ln Z_{nf}}{\partial f}$$

Noting that $\frac{de_{n}\left(x\right)}{dx}=e_{n-1}\left(x\right)$ and $e_{n}\left(x\right)=e_{n-1}\left(x\right)+\frac{x^{n}}{n!}$ yields the average position $\langle x_{p}\rangle$:

(6) $$\langle x_{p}\rangle =\left(n+1\right)k_{B}Tf^{-1}+X_{c}$$

where:

(7) $$X_{c}=-k_{B}Tf^{-1}\frac{{\left(2\beta f\lambda \right)}^{n+1}}{n!}{\left[e^{2\beta f\lambda }-e_{n}\left(2\beta f\lambda \right)\right]}^{-1}$$

This looks very different to the expression $x_{p}=nk_{B}T{\langle f\rangle }^{-1}$, valid when the piston position is the independent variable. There are two differences. The first is a minor factor of n+1 compared to *n*. This arises simply because when force is the independent variable, the piston ring itself is somewhat free to move and increases the effective number of rings by one. The second difference is the term, $X_{c}$. This is large if the force is small, *i.e.*, in practice, if $f<\left(n+1\right)k_{B}T/\left(2e\lambda \right)$, but is negligible at larger forces. This arises because of the constraint that $\langle x_{p}\rangle$ cannot be larger than 2λ.

## 3. Two Stripes with Piston Position As the Independent Variable

The previous section showed that the simple piston-rotaxane system behaves approximately as an ideal gas. In many real rotaxane systems, there are potential stations along the axle that attract the rings. The potential seen by the rings is then a function of position, and the analysis of the simple piston-rotaxane needs to be modified. We start this here by examining the simplest system possible, with two potential stripes of equal size.

We divide the axle into two equal regions of a length of *λ*, which interact differently with the mobile rings (Figure 2). The one farthest from the piston, stripe 2, has a potential $k_{B}TA$, where *A* is a constant. Each mobile ring in this region experiences an addition to its energy of $k_{B}TA$. In the other region (closest to the piston end), stripe 1, the addition to the energy is zero. In practice, since *A* can be positive or negative, and since the energy origin is arbitrary, this covers all possible scenarios of a repulsive or attractive stripe. The major limitations are that the stripe widths are equal, and the piston ring itself experiences no potential. Both of these assumptions can be readily relaxed, but we keep them in order to keep the results as simple as possible.

The algorithm for calculating the average force felt by the piston is the same as for the non-striped rotaxane. The calculations appear in the Appendix. The free energy is:

(8) $$F=\left\{\begin{array}{cc} -k_{B}T\ln\left(x_{p}e^{-A}\right) & \mathrm{if}0<x_{p}\leq \lambda \\ -k_{B}T\ln\left(\lambda e^{-A}+x_{p}-\lambda \right) & \mathrm{if}\lambda <x_{p} \end{array}\right.$$

The average force is Equation (16):

(9) $$\langle f\rangle =\left\{\begin{array}{cc} k_{B}Tx_{p}^{-1} & \mathrm{if}0<x_{p}\leq \lambda \\ k_{B}T{\left(\lambda e^{-A}+x_{p}-\lambda \right)}^{-1} & \mathrm{if}\lambda <x_{p} \end{array}\right.$$

**Figure 3:**
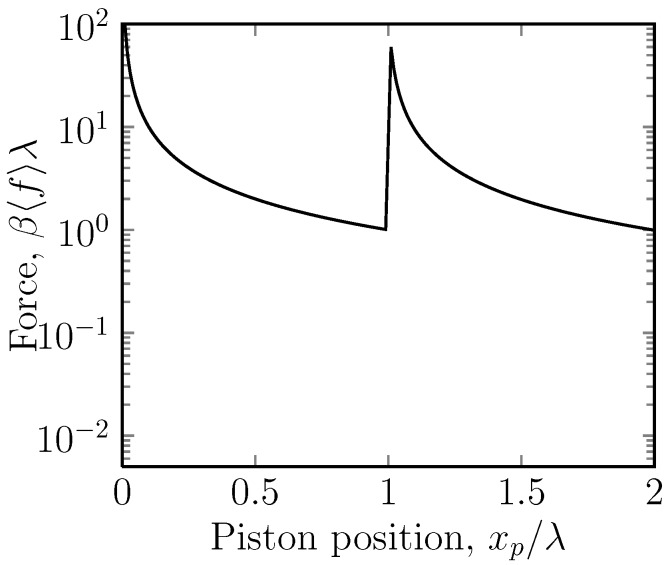
A semi-log plot of the dimensionless mean force, β〈f〉λ, *versus* the position of the piston ring, $x_{p}$, for the case of a potential $U=5\;k_{B}T$ (*i.e.*, A=5) acting on stripe 2. Stripe 2 is thus repulsive. Here, the position of the piston ring, $x_{p}$, is the independent variable and is imposed on the system by the piston. The average force, 〈f〉, of the mobile ring on this is the measured quantity. This is the case for one mobile ring. In the case of *n* mobile rings, the force is multiplied by a factor of *n*. Under gradual compression, the force gradually increases, until the interface between the stripes is reached, when a sudden decrease is observed.

As the piston ring crosses between the stripes, there is a jump in the force of $\Delta \langle f\rangle =k_{B}T\lambda^{-1}\left(e^{A}-1\right)$. This jump (Figure 3) is most easily understood in the case where A≫1. Let us assume that the piston is gradually decompressed, beginning at $x_{p}$ close to zero. As long as $x_{p}<\lambda$, all the rings are confined to stripe 2, and we are just decompressing a gas in a uniform potential. The force is that for a simple gas $\langle f\rangle =nk_{B}T/x_{p}.$ However, as soon as the piston crosses into stripe 1, some of the rings can jump into a region of much lower potential. As the piston ring crosses the stripe boundary, the free energy is continuous, but the force undergoes a discontinuous jump upwards, because the rings move from a region of a size of *λ* to one which is of a size of $x_{p}-\lambda \ll \lambda$. Further increasing of $x_{p}$ decreases the force.

What is slightly counterintuitive about this problem is that under compression, the free energy always increases, but the force, which is the negative of the slope of the free energy, is not monotonic and undergoes a discontinuous jump.

**Figure 4:**
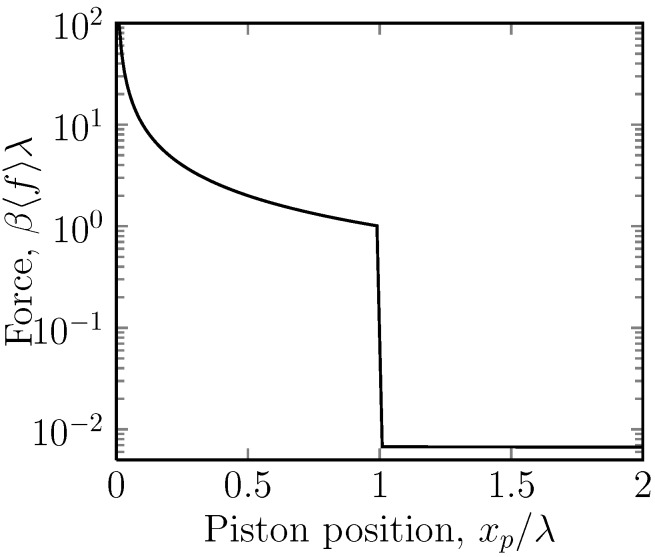
A semi-log plot of the dimensionless mean force, β〈f〉λ, *versus* the position of the piston ring, $x_{p}$, for the case of a potential $U=-5\;k_{B}T$ (*i.e.*, A=-5) acting on stripe 2. Stripe 2 is then attractive. Here, the position of the piston ring, $x_{p}$, is the independent variable and is imposed on the system by the piston. The average force, 〈f〉, of the mobile ring on this is the measured quantity. This is the case for one mobile ring. In the case of *n* mobile rings, the force is multiplied by a factor of *n*. Under gradual compression, the force gradually increases until the interface between the stripes is reached, when a sudden increase is observed.

There are in fact two cases to consider. The most interesting case is when A>0, so that under compression, one is first compressing a gas in the lower potential region. There is then a negative jump in the force under compression. In the opposite case in which A<0 (Figure 4), the initial compression is easy, since there are few particles in stripe 1, and the eventual jump in the force is positive.

**Figure 5:**
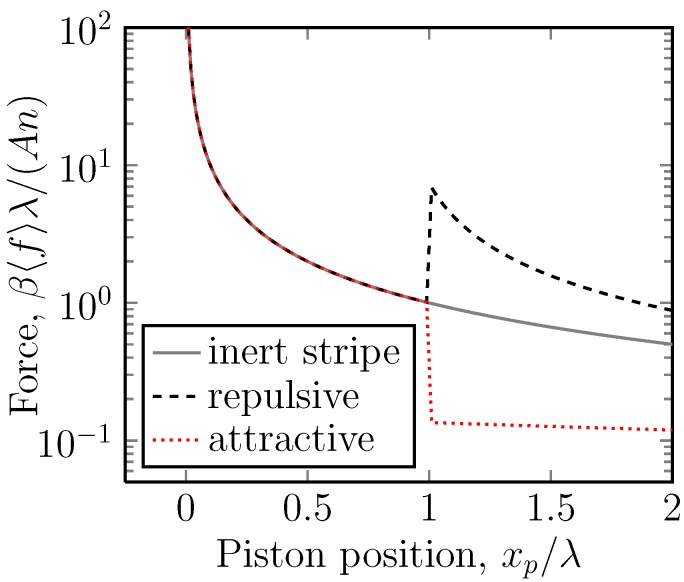
A semi-log plot of the dimensionless mean force, β〈f〉λ, *versus* the position of the piston ring, $x_{p}$, for potentials $U=-2\;k_{B}T$ (stripe 2 attractive), U=0 (both stripes equal, inert) and $U=2\;k_{B}T$ (stripe 2 repulsive). Here, the position of the piston ring, $x_{p}$, is the independent variable and is imposed on the system by the piston. The average force, 〈f〉, of the mobile ring on this is the measured quantity. This is the case for one mobile ring. In the case of *n* mobile rings, the force is multiplied by a factor of *n*. The inert system is the simple piston-rotaxane of Figure 1.

The case in which there is more than one mobile ring is relatively straightforward (see appendix). For *n* mobile rings, the average force is multiplied by *n* (Figure 5), so that Equation (21):

(10) $$\langle f\rangle =\left\{\begin{array}{cc} nk_{B}Tx_{p}^{-1} & \mathrm{if}0<x_{p}\leq \lambda \\ nk_{B}T{\left(\lambda e^{-A}+x_{p}-\lambda \right)}^{-1} & \mathrm{if}\lambda <x_{p} \end{array}\right.$$

## 4. Two Stripes with Force as the Independent Variable

We now keep the system the same as in the previous section, but apply a given force, *f*, to the piston. As shown in the Appendix, for one mobile ring, the partition function is Equation (24):

(11) $$Z_{1f}=\lambda^{2}\theta^{-2}\left[e^{-A}\left(1-e^{-\theta }-\theta e^{-2\theta }\right)+\left(e^{-\theta }-e^{-2\theta }-\theta e^{-2\theta }\right)\right]$$

where θ≡βfλ is a dimensionless measure of the force.

Again, one can get the average position by differentiating the partition function with respect to the force, Equation (25):

(12) $$\langle x_{p}\rangle =-\lambda Z_{1f}^{-1}\frac{\partial Z_{1f}}{\partial \theta }$$

**Figure 6:**
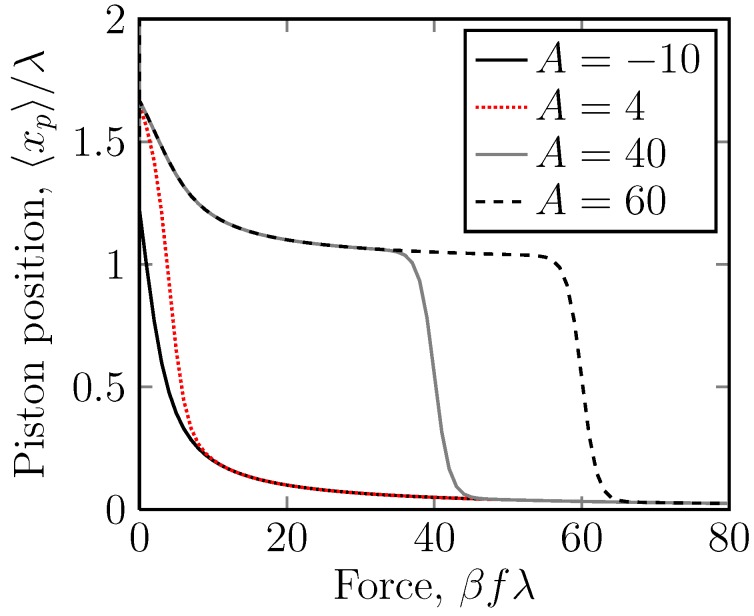
A linear plot of the dimensionless piston position, $\langle x_{p}\rangle /\lambda$, *versus* the dimensionless applied force on the piston ring, βfλ, for the case of different potentials $U=-10\;k_{B}T,4\;K_{B}T,40\;k_{B}T,60\;k_{B}T$ acting on stripe 2. Stripe 2 ranges from attractive to repulsive. Here, the position of the piston ring, $\langle x_{p}\rangle$, is the measured quantity, and the applied force on the piston, *f*, is the independent variable. This is the case for one mobile ring. Even though we only have one mobile ring, for positive A, *i.e.*, repulsive potentials, we have a sudden drop in the displacement at a critical force. The system thus exhibits yield stress behaviour.

For A>0, this produces an extension *versus* the force curve, like that shown in Figure 6. The most notable feature is a sudden drop in the extension at a critical force, *i.e.*, the system shows a yield stress. Also notable is the great difference between this curve and the equivalent curve when $x_{p}$ is the independent variable (Figure 3).

**Figure 7:**
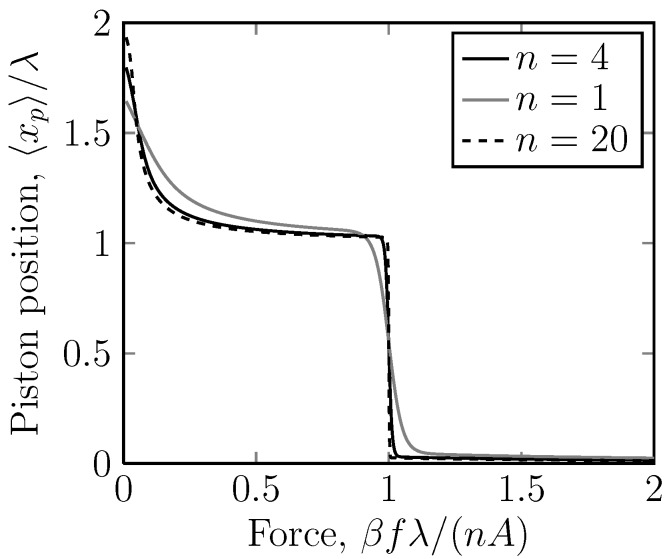
A linear plot of the dimensionless piston position, $\langle x_{p}\rangle /\lambda$, *versus* the dimensionless applied force on the piston ring, βfλ, for the case of a potential $U=40\;k_{B}T$ acting on stripe 2. Stripe 2 is thus repulsive. Here, the position of the piston ring, $\langle x_{p}\rangle$, is the measured quantity and the applied force on the piston, *f*, is the independent variable. The number of rings, n=1,4,20 is varied. Again, we have a sudden drop in the displacement at a critical force. Note, also, that the scaling, βfλ/(nA), shows that the critical force always lies at f≈nA/(βλ).

**Figure 8:**
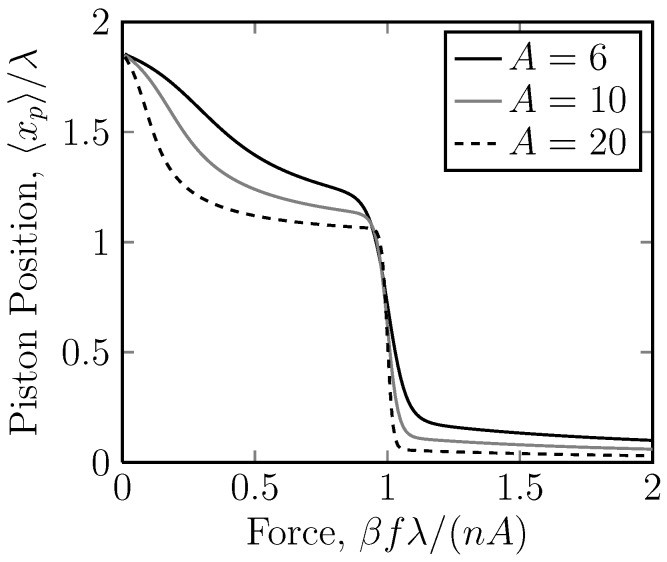
A linear plot of the dimensionless piston position, $\langle x_{p}\rangle /\lambda$, *versus* the dimensionless applied force on the piston ring, βfλ, for the case of different potentials $U=6\;k_{B}T,10\;k_{B}T,20\;k_{B}T$ acting on stripe 2. Stripe 2 is thus repulsive. Here, the position of the piston ring, $\langle x_{p}\rangle$, is the measured quantity and the applied force on the piston, and *f* is the independent variable. The number of rings is n=5. Again, we have a sudden drop in the displacement at a critical force. Note, also, that the scaling, βfλ/(nA), shows that the critical force always lies at f≈nA/(βλ).

For a system with *n* mobile rings, the calculation is even more involved, but following the Appendix, we can draw curves of $\langle x_{p}\rangle$ *versus f* for different *n* (Figure 7). As *n* gets larger, the transition becomes sharper. From Figure 7 and Figure 8, it is clear that for large *n* or *A*, the transition obeys the scaling

(13) $$f^{*}\approx k_{B}TnA\lambda^{-1}$$

The case of A<0 (Figure 6) is, as expected, less interesting. There is no sharp transition, and the extension gradually decreases as the force is increased.

## 5. Conclusions

We have reviewed some previous results for piston-rotaxanes in which the physics can be approximated by that of a one-dimensional ideal gas. We then studied what happens in the presence of a potential acting on the rings. If the potential is in the form of stripes, we can obtain sudden jumps in either the force or the displacement, depending on what is chosen as the independent variable. In particular, for the case of a given applied force, the system can exhibit a yield stress, whereby, below a critical force, the system is only weakly compressible, but for larger forces, it collapses suddenly.

Our system consists of two stripes. However, it can readily be applied to a rotaxane with many attractive stations along the backbone, provided the rings can hop between stations during the course of the experiment. There are some obvious extensions of this work to more than two stripes or to more gradually varying potentials. There would also be some interest in non-equilibrium effects.

## A. The Calculations for the Striped Piston-Rotaxane

The rotaxane is of a length of 2λ with a piston ring attached, and the distance of the piston ring from the far end of the rotaxane is $x_{p}$ (Figure 2). There are *n* mobile rings after the piston ring, and these interact with the rotaxane backbone through a potential. The potential is zero for the first half of the backbone and $Ak_{B}T$ for the second half. The piston ring is assumed to be unaffected by the potential.

There are essentially two separate experiments, the first with the piston ring position, $x_{p}$, as a given independent variable. The second has the force, *f*, on the piston ring as the independent variable.

### A.1. Given Piston Position, One Mobile Ring

Consider the case first of only one mobile ring; so, n=1. For $x_{p}<\lambda$, the mobile ring is always in the region of a potential, $Ak_{B}T$, and the partition function is then:

(14) $$Z_{1x}=\int_{0}^{x_{p}}dxe^{-\beta Ak_{B}T}=x_{p}e^{-A}$$

where $\beta \equiv \frac{1}{k_{B}T}$. If $x_{p}>\lambda$, the partition function is:

(15) $$Z_{1x}=\int_{0}^{\lambda }e^{-A}+\int_{\lambda }^{x_{p}}dxe^{0}=\lambda e^{-A}+x_{p}-\lambda$$

The free energy of the system is then $F=-k_{B}T\ln\left(Z_{1x}\right)$, and the average force on the piston ring is $\langle f\rangle =-\frac{\partial F}{\partial x_{p}}=\beta^{-1}Z_{1x}^{-1}\frac{\partial Z_{1x}}{\partial x_{p}}$.

This leads to an expression for the average force given by:

(16) $$\langle f\rangle =\left\{\begin{array}{cc} k_{B}Tx_{p}^{-1} & \mathrm{if}0<x_{p}\leq \lambda \\ k_{B}T{\left(\lambda e^{-A}+x_{p}-\lambda \right)}^{-1} & \mathrm{if}\lambda <x_{p} \end{array}\right.$$

The jump in the average force when $x_{p}$ crosses the $x_{p}=\lambda$ boundary, i.e., crosses a stripe, is then $\Delta \langle f\rangle =k_{B}T\lambda^{-1}\left(e^{A}-1\right)$.

### A.2. Given Piston Position, Many Mobile Rings

In the case of *n* mobile rings, located at $x_{i}$, with i=1...n, and subjected to potentials, $U(x_{i})$, the total energy is $U\left(x_{1}\right)+U\left(x_{2}\right)+...U\left(x_{n}\right)$. The rings cannot penetrate each other and, so, remain ordered, so that $x_{p}>x_{n}>x_{n-1}.....>x_{2}>x_{1}$. This means the partition function is:

(17) $$Z_{nx}=\int_{0}^{x_{p}}dx_{n}\int_{0}^{x_{n}}dx_{n-1}...\int_{0}^{x_{2}}dx_{1}\exp\left(-\beta \left(U\left(x_{1}\right)+U\left(x_{2}\right)+...U\left(x_{n}\right)\right)\right)$$

or

(18) $$Z_{nx}=\int_{0}^{x_{p}}dx_{n}\int_{0}^{x_{n}}dx_{n-1}...\int_{0}^{x_{2}}dx_{1}e^{-\beta U(x_{1})}e^{-\beta U(x_{2})}e^{-\beta U(x_{n})}$$

However, the particles are identical, and so, rather than integrating over the small region of phase space defined by $x_{p}>x_{n}>x_{n-1}.....>x_{2}>x_{1}$, we can integrate over the entire region, $0<x_{n}<x_{p},0<x_{n-1}<x_{p},...,0<x_{1}<x_{p}$, and, then, divide by the number of ways of swapping the particles, n!, so:

(19) $$Z_{nx}=\frac{1}{n!}\int_{0}^{x_{p}}dx_{n}\int_{0}^{x_{p}}dx_{n-1}...\int_{0}^{x_{p}}dx_{1}e^{-\beta U(x_{1})}e^{-\beta U(x_{2})}e^{-\beta U(x_{n})}$$

Each integral here is independent of the others, so:

(20) $$Z_{nx}=\frac{1}{n!}Z_{1x}^{n}$$

where $Z_{1x}$ is the partition function for a single particle. The free energy and the force for *n* particles are then just *n* times that for a single particle:

(21) $$\langle f\rangle =\left\{\begin{array}{cc} nk_{B}Tx_{p}^{-1} & \mathrm{if}0<x_{p}\leq \lambda \\ nk_{B}T{\left(\lambda e^{-A}+x_{p}-\lambda \right)}^{-1} & \mathrm{if}\lambda <x_{p} \end{array}\right.$$

### A.3. Given Applied Force, One Mobile Ring

A force, *f*, applied to the piston ring when it is located at $x_{p}$ is equivalent for a potential on the piston ring, $U_{p}=fx_{p}$; so the partition function for a given applied force *f* is:

(22) $$Z_{1f}=\int_{0}^{2\lambda }dx_{p}\int_{0}^{x_{p}}dx_{1}e^{-\beta fx_{p}}e^{-\beta U(x_{1})}$$

This can be written as:

(23) $$Z_{1f}=\int_{0}^{2\lambda }dx_{p}e^{-\beta fx_{p}}\int_{0}^{x_{p}}dx_{1}e^{-\beta U(x_{1})}=\int_{0}^{2\lambda }dx_{p}e^{-\beta fx_{p}}Z_{1x}\left(x_{p}\right)$$

where $Z_{1x}\left(x_{p}\right)$ is the partition function for a single particle for the case when $x_{p}$ is the independent variable.

$Z_{1f}$ can be evaluated by integration to give

(24) $$Z_{1f}=\lambda^{2}\theta^{-2}\left[e^{-A}\left(1-e^{-\theta }-\theta e^{-2\theta }\right)+\left(e^{-\theta }-e^{-2\theta }-\theta e^{-2\theta }\right)\right]$$

where θ≡βfλ is a dimensionless measure of the force.

The mean displacement of the piston ring can be evaluated from $Z_{1f}$ as:

(25) $$\langle x_{p}\rangle =-\beta^{-1}\frac{\partial \ln Z_{1f}}{\partial f}=-\beta^{-1}Z_{1f}^{-1}\frac{\partial Z_{1f}}{\partial f}=-\lambda Z_{1f}^{-1}\frac{\partial Z_{1f}}{\partial \theta }=$$

(26) $$\frac{\lambda \;\left(\theta \;e^{-\theta -A}-\theta \;e^{-\theta }+2\;e^{-2\;\theta -A}\theta^{2}+e^{-2\;\theta -A}\theta +3\;e^{-2\;\theta }\theta +2\;e^{-2\;\theta }\theta^{2}-2\;e^{-A}+2\;e^{-\theta -A}-2\;e^{-\theta }+2\;e^{-2\;\theta }\right)}{\theta \;\left(-e^{-A}+e^{-\theta -A}-e^{-\theta }+e^{-2\;\theta -A}\theta +e^{-2\;\theta }+e^{-2\;\theta }\theta \right)}$$

### A.4. Given Force, Many Mobile Rings

For a given force, *f*, acting on the piston rings and *n* mobile rings, the partition function can be written in terms of an integral of $Z_{nx}\left(x_{p}\right)$ as follows:

(27) $$Z_{nf}=\int_{0}^{2\lambda }dx_{p}e^{-\beta fx_{p}}Z_{nx}\left(x_{p}\right)=\frac{1}{n!}\int_{0}^{2\lambda }dx_{p}e^{-\beta fx_{p}}Z_{1x}^{n}\left(x_{p}\right)$$

The calculation is somewhat lengthy. The result is:

(28) $$Z_{nf}=\lambda^{n+1}\theta^{-(n+1)}\left[e^{-An}-e^{-An-\theta }e_{n}\left(\theta \right)+e^{-\theta }e_{n}\left(e^{-A}\theta \right)-e^{-2\theta }e_{n}\left(\theta \left(1+e^{-A}\right)\right)\right]$$

where:

(29) $$e_{n}\left(x\right)\equiv \sum_{k=0}^{n}\frac{x^{k}}{k!}$$

It is easy to check that this reproduces the n=1 case correctly.

Again, we can obtain the piston ring displacement from:

(30) $$\langle x_{p}\rangle =-\lambda Z_{nf}^{-1}\frac{\partial Z_{nf}}{\partial \theta }$$

although we do not attempt to write down the result explicitly.
